# PolyRad – Protection Against Free Radical Damage

**DOI:** 10.1038/s41598-020-65247-y

**Published:** 2020-05-20

**Authors:** Hannah Kim, Yin Tse, Andrew Webb, Ethan Mudd, Muhammad Raisul Abedin, Melanie Mormile, Subhadeep Dutta, Kaushal Rege, Sutapa Barua

**Affiliations:** 1 Department of Biological Sciences, Missouri University of Science and Technology, Rolla MO, 65409 USA; 2 Department of Chemical and Biochemical Engineering, Missouri University of Science and Technology, Rolla MO, 65409 USA; 3 School of Molecular Sciences, Arizona State University, Tempe, AZ 85287 USA; 4 Chemical Engineering, School for Engineering of Matter, Transport and Energy, Arizona State University, Tempe, AZ 85287 USA

**Keywords:** Engineering, Chemical engineering

## Abstract

The effects of elevated levels of radiation contribute to the instability of pharmaceutical formulations in space compared to those on earth. Existing technologies are ineffective at maintaining the therapeutic efficacies of drugs in space. Thus, there is an urgent need to develop novel space-hardy formulations for preserving the stability and efficacy of drug formulations. This work aims to develop a novel approach for the protection of space pharmaceutical drug molecules from the radiation-induced damage to help extend or at least preserve their structural integrity and potency. To achieve this, free radical scavenging antioxidant, Trolox was conjugated on the surface of poly-lactic-co-glycolic acid (PLGA) nanoparticles for the protection of a candidate drug, melatonin that is used as a sleep aid medication in International Space Station (ISS). Melatonin-PLGA-PLL-Trolox nanoparticle as named as PolyRad was synthesized employing single oil in water (o/w) emulsion solvent evaporation method. PolyRad is spherical in shape and has an average diameter of ~600 nm with a low polydispersity index of 0.2. PolyRad and free melatonin (control) were irradiated by UV light after being exposed to a strong oxidant, hydrogen peroxide (H_2_O_2_). Bare melatonin lost ~80% of the active structure of the drug following irradiation with UV light or treatment with H_2_O_2_. In contrast, PolyRad protected >80% of the active structure of melatonin. The ability of PolyRad to protect melatonin structure was also carried out using 0, 1, 5 and 10 Gy gamma radiation. Gamma irradiation showed >98% active structures of melatonin encapsulated in PolyRads. Drug release and effectiveness of melatonin using PolyRad were evaluated on human umbilical vein endothelial cells (HUVEC) *in vitro*. Non-irradiated PolyRad demonstrated maximum drug release of ~70% after 72 h, while UV-irradiated and H_2_O_2_-treated PolyRad showed a maximum drug release of ~85%. Cytotoxicity of melatonin was carried out using both live/dead and MTT assays. Melatonin, non-radiated PolyRad and irradiated PolyRad inhibited the viability of HUVEC in a dose-dependent manner. Cell viability of melatonin, PolyRad alone without melatonin (PolyRad carrier control), non-radiated PolyRad, and irradiated PolyRad were ~98, 87, 75 and 70%, respectively at a concentration $\sim$ 0.01 ${mg}/{ml}$ ($10\mu g/{ml}$). Taken together, PolyRad nanoparticle provides an attractive formulation platform for preventing damage to pharmaceutical drugs in potential space mission applications.

## Introduction

As NASA prepares for exploration missions beyond low Earth orbit with no opportunities for resupply, pharmaceutical instability may present significant risk to the crew health due to the loss of efficacy over time. Astronauts suffer from sleep disruption during space flight that can affect their mental and physical health in performing routine work^1–4^. Therefore, mitigation against the effects of space radiation on pharmaceutical drugs is necessary for the success of long-term space missions. NASA is primarily concerned with the health risks for astronaut exposure to radiation and other flight infections. NASA estimates that a 30-month mission to Mars would expose astronauts to over 900 millisieverts (mSv) (1000 mSv = 1 gray), far beyond safe or acceptable *Occupational Safety and Health Administration (*OSHA) limits of 50 mSv in the United States^5,6^. Persistent radiation induces oxidative stress associated with the central nervous system, blood coagulation abnormalities, fibrosis, heart failures, gene expression changes, cancer as long-term effects and other physiological abnormalities^7–10^.

High energy radiation and oxidative stress due to UV and gamma radiation induce radiolysis of water generating highly reactive hydrogen (H•) and hydroxyl (OH•) radicals that diffuse to other molecules and react with particular chemical groups resulting in a loss of biochemical activity^11^. There are three mechanisms of radiation damage caused by OH• in the chemical structure of a compound: the abstraction of $-H$ by OH• leaving an unpaired electron on the carbon atom, the formation of a hydroxy derivative by breaking double bonds (*e.g*., $-C=C-$) and the electron transfer from anionic groups (*e.g*., ${{Cl}}^{-}$) to OH•^11^. The production of reactive OH• has been demonstrated by chemical means such as UV photolysis of hydrogen peroxide (H_2_O_2_) and gamma irradiation using colbalt-60 (^60^Co) or cesium 137 (^137^Cs)^12–17^. The effects of radiation on a variety of pharmaceutical drug molecules have been studied using either gamma radiation or H_2_O_2_ hydrolysis by UV light at different doses that show linear proportionality of the decreasing amount of active substances with the dose of radiation^18–22^.

One way to countermeasure the adverse effects of radiation is by creating an artificial shield. Numerous experimental investigations and theoretical studies have reported the use of a variety of shielding materials that include concrete, polymer composites, heavy metals such as lead, composites of lead oxide, tungsten and tin, *etc*. for attenuation and absorption of the undesired radiations^23–25^. However, the materials are heavy and bulky that add unwanted features to most spacecraft applications. Materials with lightweight and low volumes are preferred due to space and maneuverability constraints imposed by the vehicles^26^. Polymers are preferred for their radiation resistant and neutron shielding capabilities^27–32^, however, their mechanical and thermal stability can be compromised by the heat generated from irradiation^27^. A combination of metal and polymer microparticles and nanoparticles using radiation-resistant polymer has shown to improve the radiation-resistance properties of the composite material^28,33–42^. Based on the previous studies by others, it is hypothesized that polymer nanoparticles can be designed to minimize the radiation induced damage by scavenging free radicals with the use of antioxidants. Other medical countermeasures including small molecule antioxidants, such as radioprotective agents such as melatonin, Trolox, vitamin C, vitamin E, cysteine and others, have also been used as radioprotective agents^43–46^.

**Figure 1:**
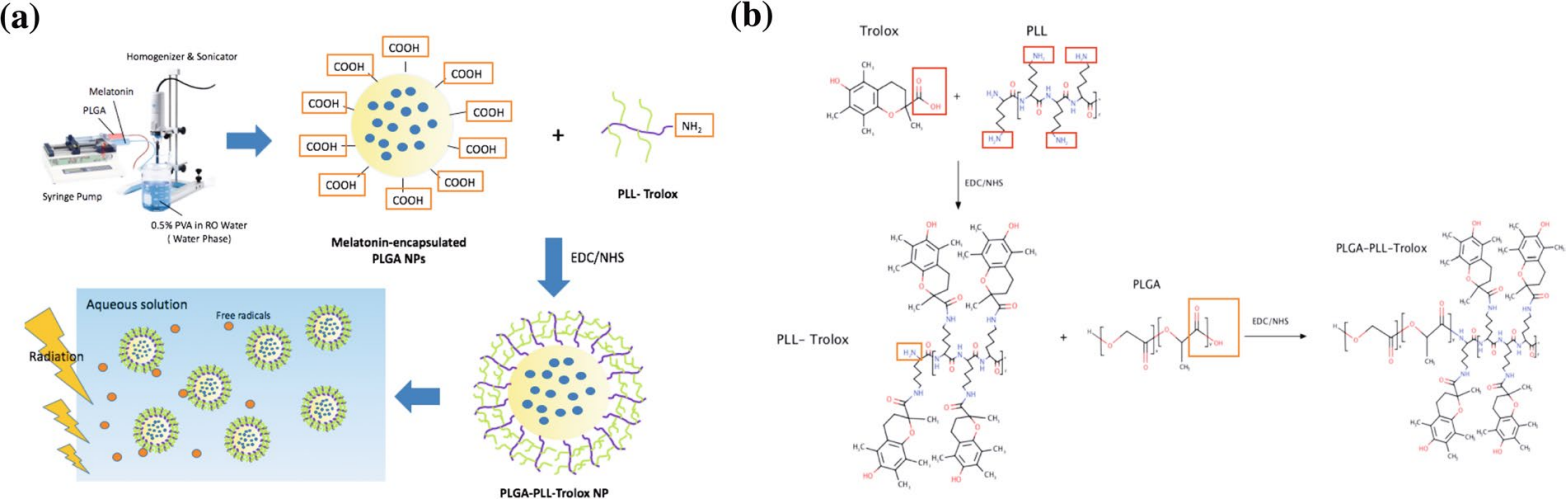
Design of PolyRad with the core of PLGA particles conjugated with PLL and Trolox. (**a**) Schematic of solvent diffusion method to synthesize melatonin encapsulated PLGA nanoparticles. **(b)** The surface of the particles was conjugated with PLL-Trolox conjugates which were synthesized separately in a parallel reaction. It is hypothesized that the nanoparticle formulation protects melatonin from photochemical degradation.

The objective of this work is to engineer a self-protected medication using a polymeric nanoparticle assembly of a biocompatible polymer, poly (lactic-*co*-glycolic acid) (PLGA), a free radical scavenger molecule (Trolox) and a linker poly-*l*-lysine (PLL) (Fig. 1a). Melatonin is used as a model relevant to space flight missions. In this design, melatonin was encapsulated in the core of PLGA polymer nanoparticles using an oil/water single emulsion solvent evaporation method. In a separate reaction, Trolox was conjugated to PLL *via* EDC/NHS chemistry for conjugating the carboxylates in Trolox with the amines in PLL (Fig. 1b). The residual amine groups on PLL-Trolox were further conjugated on the surface of melatonin-loaded PLGA nanoparticles *via* the same process of EDC/NHS reaction. It is hypothesized that Trolox conjugation on the surface of nanoparticles (PolyRad) protects the chemical stability of melatonin during spaceflight mimicking conditions that generate excessive radiation-induced free radicals through its potential for antioxidant capacity and free radical scavenging activity. We further hypothesize that the PolyRad approach offers great benefits by increasing the specific surface area and accelerating the dissolution velocities of drug molecules, which ultimately has a beneficial impact on the drug release and associated bioavailability.

## Materials and Methods

### Materials

All reagents were purchased from Sigma-Aldrich (St. Louis, MO, USA) unless otherwise specified.

### Synthesis of PolyRad nanoparticles

Melatonin-PLGA-PLL-Trolox (PolyRad) nanoparticles were prepared in three steps: entrapment of melatonin in PLGA (melatonin-PLGA), conjugation of Trolox with PLL (PLL-Trolox), and surface conjugation of the PLL-Trolox on melatonin-PLGA nanoparticles (melatonin-PLGA-PLL-Trolox). Melatonin (N-acetyl-5-methoxytryptamine) was encapsulated in PLGA polymer using water-in-oil (w/o) emulsion phase separation method (Fig. 1a)^47–49^. Briefly, 1 ml of each 1% (w/v) of melatonin dissolved in DMSO and 5% (w/v) of PLGA polymer dissolved in dichloromethane (DCM) were added to 20 ml of 1% (w/v) polyvinyl alcohol (PVA) solution using a syringe pump. The flow rate of 1 ml/h was maintained under sonication and homogenization. The nanoparticle suspension was stirred (350 rpm) overnight (for about 18 h) to remove DCM by evaporation.

Conjugation of Trolox to PLL was carried out by activation of carboxyl groups in Trolox followed by reaction with the primary amine groups of PLL polymer resulting in the formation of amide bonds (Fig. 1b). Briefly, 4 mg of 1-ethyl-3-3-dimethylaminopropyl-carbodiimide (EDC) was directly added to 1 ml of 10 mg/ml Trolox dissolved in phosphate-buffered saline (PBS) of pH 7.4. Immediately, 6 mg of N-hydroxysuccinimide (NHS) was added to the reaction. The reaction solution was mixed gently, maintained at pH 7.4, and left at room temperature (R.T.; 25 °C) to react for 15 min. PLL (11.5 mg) was added to the solution that was incubated at R.T. for 2 h while shaking (450 rpm). The residual primary amine groups of PLL-Trolox were conjugated to the surface of melatonin-PLGA nanoparticles using EDC/NHS chemistry. The final reaction solution was filtered using 100 kDa Amicon Ultra-4 centrifugal filters to remove any excess reagents.

### PolyRad characterization: size, surface charge, and melatonin encapsulation efficiency

The size and surface charge of particles before and after PLL-Trolox conjugation on melatonin encapsulated PLGA nanoparticles were measured using dynamic light scattering (DLS) and zeta potential measurements, respectively using a NanoSeries Zetasizer ZS 90 (Malvern). Samples were prepared in deionized (DI) water. Encapsulation of melatonin in PolyRad nanoparticles was assessed by measuring the absorbance at 300 nm using a microplate reader (BioTek Synergy 2) and a melatonin standard curve (S.I. Figure 1). The percentage of melatonin encapsulation capacity was calculated using the following Eq. 1:

1 $$ \% \,encapsulation\,efficiency=\frac{{M}_{0}-({M}_{u}-{M}_{s})}{{M}_{0}}\times 100$$

where, M_0_ = Initial melatonin mass, *M*_*u*_ = Melatonin mass in unwashed suspension and *M*_*s*_ = Melatonin mass in supernatant$.$

### **Characterization of PLGA-PLL-Trolox formulation** using **Fourier Transform Infrared (FT-IR) Spectroscopy**

FTIR spectroscopy was used to confirm the structural changes that occurred after PLL-Trolox conjugation with PLGA nanoparticles. Samples for FT-IR spectroscopy were prepared by mixing and grinding freeze-dried nanoparticles with FT-IR grade potassium bromide (KBr; Alpha Aesar) (w:w ratio of 1:100) to make pulverized pellets. FT-IR absorption spectra were obtained using a Thermo Nicolet NEXUS 470 FTIR instrument in the wavelength range from 4000 to 400 cm^−1^ at a resolution of 4 cm^−1^ using 32 scans per sample. The effect of background noise from the ambient air without a sample in place was subtracted from the sample spectra. All spectra were analyzed using EZ OMNIC E.S.P v.5.1 software (Thermo Scientific). The resulting FTIR spectra of PLGA-PLL-Trolox were compared to individual spectra of PLGA, PLL, and Trolox to characterize the conjugation reaction based on the intensity or shift of the vibrational bands.

### **Determination of the composition of melatonin-PLGA-PLL-Trolox (PolyRad) nanoparticles using**^**1**^**H-NMR spectroscopy**

For ^1^H-NMR spectroscopy, 10 mg of PolyRad nanoparticles, melatonin alone, PLGA alone, PLL alone, and Trolox alone were dissolved in 750 μl of DMSO-d6 except for PLGA, which was dissolved in d-Chloroform. Each solvent was used as the internal reference to determine chemical shifts (δ) in ppm. ^1^H-NMR spectra were then recorded using Bruker advanced III 400 MHz Liquid-State NMR instrument at R.T.

### Fluorometric Hydroxyl Radical Scavenging Capacity (HOSC) assay

A fluorometric HOSC assay was applied to determine the mechanisms of highly reactive free radical absorption by PLGA-PLL-Trolox composite and Trolox alone was used as a standard^50^. OH• radicals were generated using Fe (III)/H_2_O_2_ Fenton-like reaction system in a pH 7.4 sodium phosphate buffer^51^. Assay reactions were carried out in Costar black 96-well polystyrene plates and analyzed using a microplate reader (BioTek Synergy 2) with an excitation wavelength of 485 nm, emission wavelength of 530 nm, and 0.1 s read time for each well with each plate read once per minute for 3 h. The reaction mixture contained 170 μl of 9.28 × 10^−8^ M fluorescein (FL) prepared in 75 mM sodium phosphate buffer (pH 7.4), 30 μl of blank or antioxidant, 40 μl of 0.1990 M H_2_O_2_ and 60 μl of 3.43 mM FeCl_3_ added in that order^50^. Trolox concentrations of 20, 40, 60, 80, and 100 μM were used. Relative fluorescence intensity of each Trolox standard concentrations were calculated by dividing individual fluorescence readings by the initial fluorescence reading at time equal to zero.

### Photochemical degradation of melatonin

To examine the resistance to photochemical degradation of melatonin, 2 mg of PolyRads containing 2.5 μg melatonin were dissolved in 1 ml 40% acetonitrile-60% water (v/v) solvent and spiked with 0.75 mM hydrogen peroxide (H_2_O_2_) followed by UV radiation at 254 nm and 39 W spectral output for 0, 1, 2, 4, 6 and 8 h. H_2_O_2_ is known to be decomposed into hydroxyl radical (OH•) and superoxide ions (${O}_{2}^{-{\boldsymbol{.}}}$) when exposed to UV radiation. Pure melatonin powder was used as a control and were treated with 0.75 mM H_2_O_2_ followed by UV irradiation at every 20 min for up to 2 h. The reaction progress was monitored by determination of melatonin concentration and its two degradant products (*N*^1^-acetyl-*N*^2^-formyl-5-methoxykynuramine, AFMK and 6-hydroxymelatonin) using Agilent 1260 Liquid Chromatography (LC) High-Performance Liquid Chromatography (HPLC) apparatus equipped with Waters C18 column of 4.6 × 250 mm (particle size 5 μm). The system consisted of a vacuum degasser, a binary pump unit, an autosampler for sample injections, a thermostat column compartment, and a photodiode array detector (DAD) for peak analysis at 304 nm. The mobile phase used was acetonitrile −25 mM ammonium acetate (40:60, v/v). The system was run at ambient temperature (~22 °C) with a flow rate of 1.0 ml/min. Sample injections of 30 μl were taken and observed for characteristic peaks over 8 min intervals between injections. Quantification of melatonin and degradation products were carried out using calibration curves obtained by repeated injections of standard solutions of known concentration.

### Gamma irradiation of PolyRad

The effects of gamma irradiation on the chemical structure of melatonin were carried out using radioactive cobalt 60 (^60^Co) isotope of activity 200 mCi. Two sample groups of melatonin alone and PolyRad were prepared in 1 ml of 10 mg/ml aqueous solutions and irradiated at 1, 5 and 10 Gray (Gy) doses. Non-radiated (0 Gy) melatonin and nanoparticles were used as controls. The samples were lyophilized and dissolved in DMSO-d6. The effects of gamma irradiation on the structure of melatonin alone and melatonin in PolyRad nanoparticles were investigated using ^1^H-NMR spectroscopy (Bruker instrument 400 MHz). The spectra were calibrated and normalized relative to DMSO-d6 at 25 °C (δ = 0 ppm). After ^1^H-NMR measurements, the relative area under the curves were calculated with respect to non-radiated melatonin standards and plotted as a function of radiation doses.

### ***In vitro*drug release kinetics**

*In vitro* release of melatonin from non-irradiated and irradiated PolyRad was carried out in PBS at pH 7.4. Each 10 mg of PolyRad and melatonin powder were put in separate 1.5 ml centrifuge tubes and exposed to 0.75 mM H_2_O_2_ followed by UV irradiation for 2 h. PolyRad was suspended in 10 ml PBS and kept in a 37 °C water bath. PBS solution of 500 μl was withdrawn at 0, 5, 10, 15 and 30 min and then at 1, 2, 4, 8, 12, 24, 48 and 72 h. The solution was centrifuged at 1000 rcf for 1 min. The supernatant (150 μl) was put in a 96 well assay plate in triplicates. PBS was used as a blank. Melatonin concentrations were measured using absorbance at 300 nm using the microplate reader and melatonin standard curve. The drug release kinetics data were plotted as log cumulative drug release *versus* log time (Eq. 2).

2 $$\log ({M}_{(t-\tau )}/{M}_{\infty }-b)=m\,\log (t-\tau )+\,\log \,k$$

where ${M}_{(t-\tau )}$ is the cumulative drug release at time $t$, ${M}_{\infty }$ is the cumulative drug release at infinite time, ${M}_{(t-\tau )}/{M}_{\infty }$ is the fractional drug release at time $t$, $\tau$ is the lag time, $b$ is the fractional drug burst release, $k$ is a kinetic constant that measures the drug release rate characteristic of the drug/polymer system, and $n$ is the release exponent which characterizes the drug release mechanism^52–58^.

### Melatonin cytotoxicity

To determine and compare the cytotoxicity of melatonin on endothelial cells, human umbilical vein endothelial cells (HUVEC) were treated with free melatonin, melatonin-loaded PolyRad (carrier control), PolyRad, PolyRad treated with 10 Gy of gamma radiation, and PolyRad treated with 0.75 mM of H_2_O_2_ followed by 1 h of UV radiation. HUVEC were seeded in Costar 96 well assay plate (sterile, black, clear-bottom with lid) at a cell density of 10,000 cells/well. The cells were incubated in PromoCell medium with 10% FBS and 1% penicillin-streptomycin (10,000 units/ml) at a 37 °C and 5% CO_2_ incubator. Cells were allowed to grow overnight. The cells were incubated for 72 h with melatonin solution control and PolyRad at melatonin concentrations of 0.01, 0.1, 1 and 10 mg/ml. PBS was used as a negative control. Untreated viable (live) HUVEC were used as a positive control. After 72 h, cells were rinsed with PBS and cellular viability were measured using MTT assay (Invitrogen). The reduction of MTT to formazan by viable HUVEC was quantified measuring absorbance at 570 nm. Absorbance of treated cells were measured and calculated by subtracting the mean background level of wells containing the medium. The % cell viability was calculated as absorbance ratio of treated cells to live cells as shown in Eq. (3):

3 $$ \% \,{\rm{cell}}\,{\rm{viability}}=\frac{{A}_{570{\rm{of}}{\rm{sample}}}-{A}_{570{\rm{of}}{\rm{medium}}}}{{A}_{570{\rm{of}}{\rm{live}}{\rm{cells}}}-{A}_{570{\rm{of}}{\rm{medium}}}}\times 100$$

To visualize cells under fluorescence microscope, live and dead cells were stained using 2 μM calcein AM and 4 μM of EthD-1, respectively following the manufacturer’s protocol. Images were acquired using a 10X Plan Fluor (NA = 0.3) objective equipped with a Carl Zeiss Axio Observer Z1 microscope at excitation/emissions of 470 ± 40/525 ± 50 and 545 ± 25/605 ± 70, respectively.

## Results

### Characterization of PolyRad

**Figure 2:**
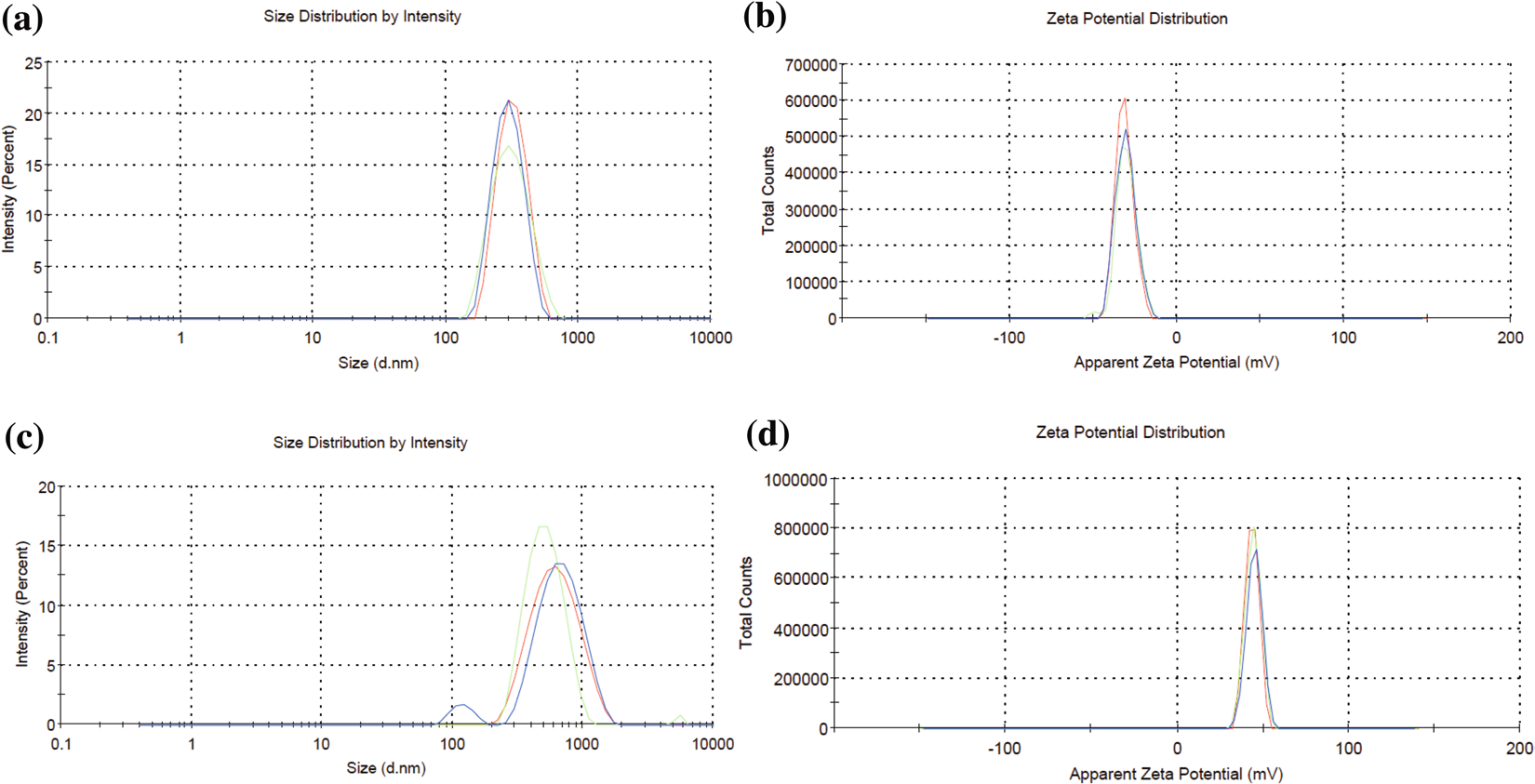
Characterization of PolyRad nanoparticles. (**a**) Size of melatonin encapsulated PLGA nanoparticles; **(b)** Zeta potential of melatonin encapsulated PLGA nanoparticles; **(c)** size of melatonin-PLGA-PLL-Trolox conjugates; and **(d)** Zeta potential of melatonin-PLGA-PLL-Trolox conjugates. The three colors in each graph represent three independent experiments.

We synthesized melatonin-encapsulated PLGA nanoparticles with an average diameter of 325 ± 21 nm (Fig. 2a), polydispersity index (pDI) of 0.07 ± 0.05, and a zeta potential of about −23 ± 8.5 mV (Fig. 2b) as confirmed by the DLS analysis. The PLL-Trolox shell attributed to the increase in particle size due to the deposition of its complex onto the surface of melatonin-PLGA nanoparticles. The average diameter of PLL-Trolox conjugated PolyRad nanoparticles were approximately 90% larger (622 ± 114 nm; Fig. 2c) than melatonin-PLGA control nanoparticles without PLL-Trolox shell as seen by the shift in the average peak intensity to the right-hand side. The pDI and zeta potential increased to 0.23 ± 0.12 and 29 ± 10 mV (Fig. 2d), respectively, indicating the successful conjugation of positively charged PLL on the surface of nanoparticles. The encapsulation efficiency of melatonin inside nanoparticles was determined as 44 ± 12% (Table 1).

**Table 1:** Percent encapsulation efficiency of melatonin in PolyRad nanoparticles.

Initial mass of melatonin, ${M}_{0}$ (μg)	Melatonin concentration in unwashed suspension, ${M}_{{\rm{u}}}$ (μg/ml)	Melatonin concentration in supernatant, ${M}_{{\rm{s}}}$ (μg/ml)	Encapsulated melatonin, ${M}_{0}-({M}_{u}-{M}_{s})$ (μg)	Encapsulation efficiency (%)
7500	200 ± 46	122 ± 54	4410 ± 1261	44 ± 12

### Fourier-Transform Infrared Spectroscopy (FT-IR) for the determination of PLL and Trolox conjugation to PLGA nanoparticles

**Figure 3:**
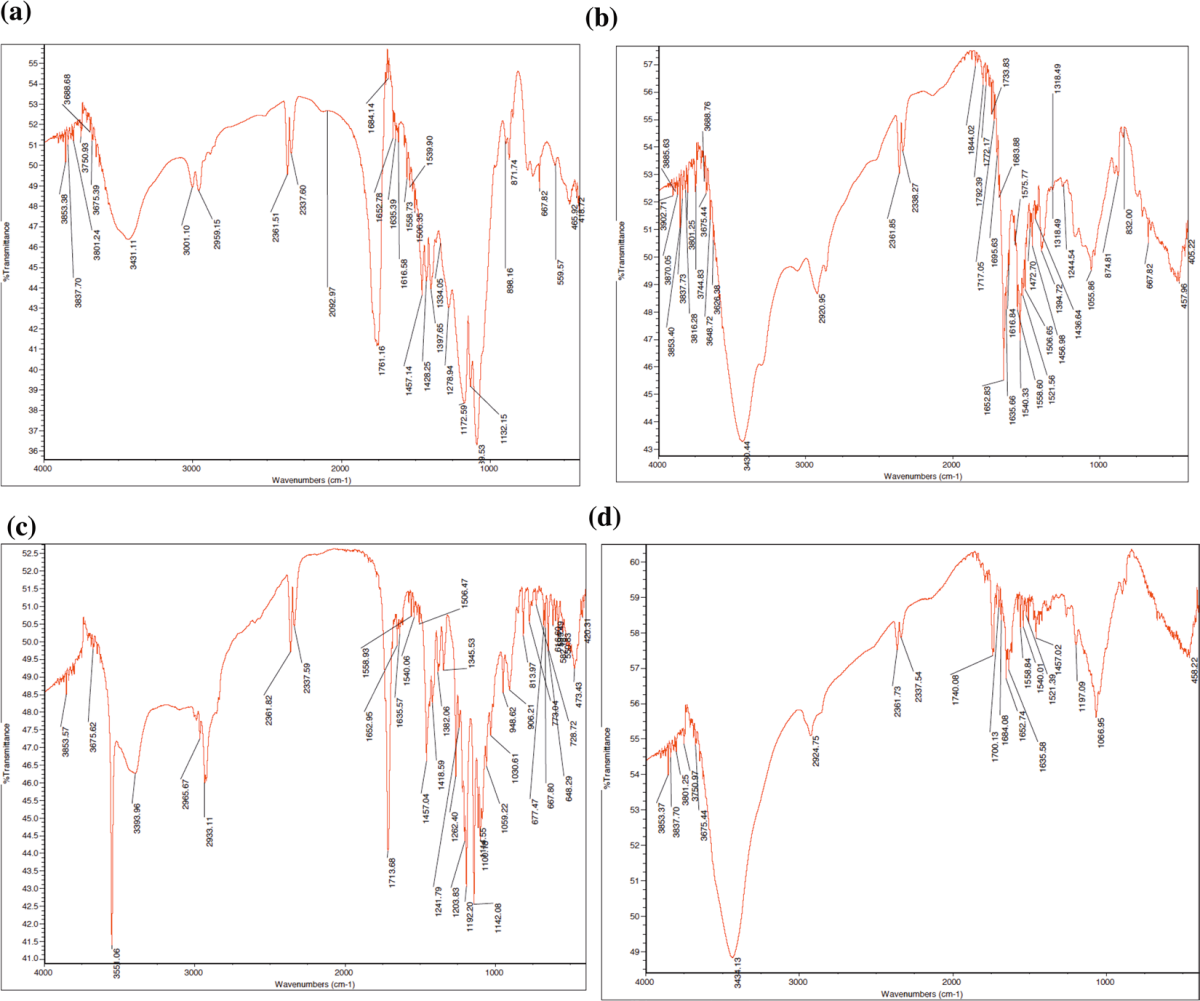
FTIR spectroscopy for investigation of melatonin-PLGA-PLL-Trolox nanoparticles. FTIR spectra analysis was performed to confirm the conjugation of PLL and Trolox to the surface of PLGA nanoparticles. **(a)** PLGA alone, **(b)** PLL alone, **(c)** Trolox and **(d)** PLGA-PLL-Trolox nanoparticles.

PLL-Trolox conjugation to PLGA nanoparticles was accomplished by reacting amine groups of PLL with carboxyl groups (-COOH) in PLGA. FTIR studies were carried out to characterize the related structural modifications (Fig. 3). PLGA spectra’s absorbance peaks appear strong at 1,765–1,720 cm^−1^ for ester carbonyl and carboxyl C=O stretch, ether C-O-C stretch absorbance peak at 1,290–1,180 cm^−1^, and secondary alcohol C-O stretch at 1,124–1,087 cm^−1^ (Fig. 3a). Broad medium absorbance peak of PLGA alkyl C-H stretch at 3,000–2,840 cm^−1^ and alcohol O-H at 3,550–3,200 cm^−1^. PLL’s spectra present strong amide C=O stretch peak at 1,680–1,640 cm^−1^, a broad, strong primary and secondary N-H stretch peak at 3,500–3,250 cm^−1^ and 3,350–3,310 cm^−1^, respectively (Fig. 3b). Also, a broad medium bend of PLL alkyl C-H stretch peak and amine N-H stretch appear correspondingly at 3,000–2,840 cm^−1^ and 1,650–1,540 cm^−1^. Trolox spectra shows strong phenol O-H peak at 3,550–3,200 cm^−1^ and carboxylic C=O stretch peak at 1,720–1,706 cm^−1^ (Fig. 3c). Numerous ether C-O-C peaks at 1,300–1,000 cm^−1^ and a broad, medium carboxylic O-H stretch peak at 3,300–2,500 cm^−1^ were observed. PLGA-PLL-Trolox composite spectrum displays strong secondary N-H, medium alkyl C-H, and amide C=O stretch peaks from PLL and strong phenol O-H, ether C-O-C stretch peaks from Trolox, and ester carbonyl C=O stretch and secondary alcohol C-O stretch peaks from PLGA, confirming the presence of three blocks in the synthesized composite (Fig. 3d).

### **Confirmation of the composition of PolyRad nanoparticles using**^**1**^**H NMR Assay**

**Figure 4:**
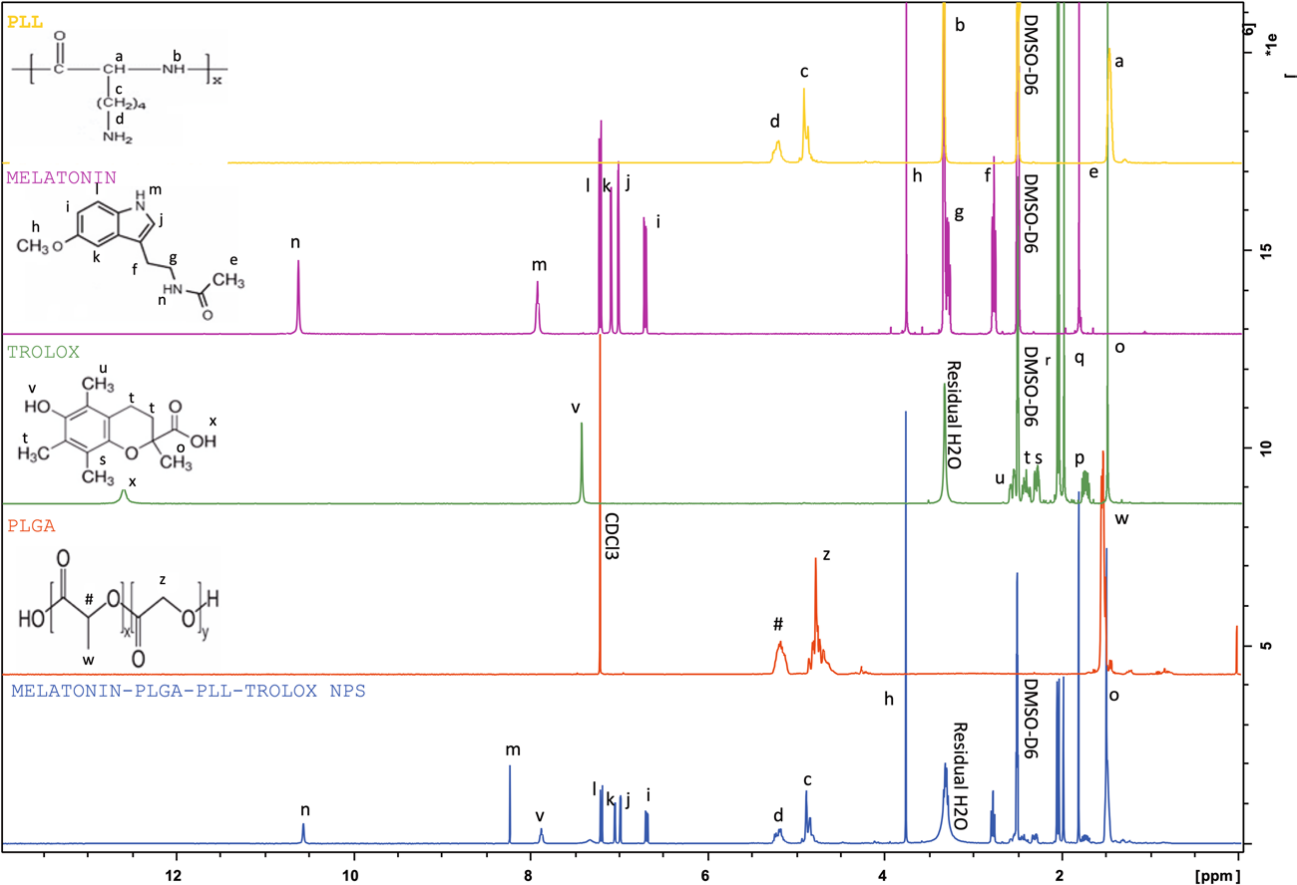
^1^H-NMR to characterize melatonin, PLGA, PLL and Trolox in PolyRad nanoparticles. ^1^H-NMR spectra acquired at room temperature for PLL, melatonin, Trolox, PLGA and melatonin-PLGA-PLL-Trolox nanoparticles to confirm the nanoparticle structure.

Formation of melatonin-PLGA-PLL-Trolox nanoparticles was evaluated using ^1^H-NMR (Fig. 4). The NMR results depicted a successful conjugation of PLL-Trolox on melatonin encapsulated PLGA nanoparticles to form PolyRad nanoparticles compared to their independent control NMR peaks (melatonin, PLGA, PLL, and Trolox alone). In Fig. 4, proton peaks at 2.5 and 7.25 ppm represent solvent background signals from DMSO-d6 and d-Chloroform, while the broad peak at 3.25 ppm represents residue water proton peak. Proton peaks from PLL, denoted by a, b, c and d which correspond to -CH-, -NH-, -CH_2_-, and -NH_2_ groups occurred at 1.7, 3.5, 4.75 and 5.2 ppm, respectively. Also, melatonin distinctive peaks denoted by letters ‘e-n’ correspond to the -CH_3_-, the two -CH_2_-, -CH_3_O-, the three phenyl -CH-, the cyclic -CH-, and the two -NH- respectively. These peaks were recorded at 1.8, 2.75, 3.25, 3.75, 6.75–7.25, 7.25, 7.8 and 11.6 ppm, respectively. For Trolox, distinctive peaks were lettered ‘o-v’. Trolox ^1^H-NMR peaks showed propionic acid -CH_3_- (o) and -OH- (p) protons at 1.5 and 1.75 ppm respectively, cyclic -CH_2_- (q and r) at 1.9–2.1 ppm, phenyl methane protons (s, t and u) at 2.0–2.2 and 2.6 ppm, and the phenyl -OH- (v) protons at 7.25 ppm. Proton peaks for PLGA were labeled from ‘w-z’ and then ‘#’. PLGA proton peaks displayed lactide -CH_3_- (w), -CH- (y), and glycolide -CH_2_- (x) protons at 1.5, 6.2 and 5.5 ppm respectively. However, the lactide end and the glycolide end -OH- protons (z and # respectively) in PLGA were missing probably by being lost to neighboring protons. Comparing with the ^1^H-NMR of the individual components, ^1^H-NMR of nanoparticles displayed virtually all the peaks that were shown in the individual components as shown in Fig. 4. However, the chemical shift of protons (b) in PLL formed an amide bond with the lactide end -OH- protons (z) of PLGA, hence we had that this amide bond occurred around 3–3.5 ppm in the NPs’ ^1^H-NMR. Likewise, proton peak at 5.25 ppm in the NPs’ ^1^H-NMR corresponds to the -NH- group of the corresponding amide bond (CONH) between primary amino group of PLL (d) and the carboxyl group of Trolox (p).

### Scavenging capacity of Trolox using fluorometric HOSC assay

**Figure 5:**
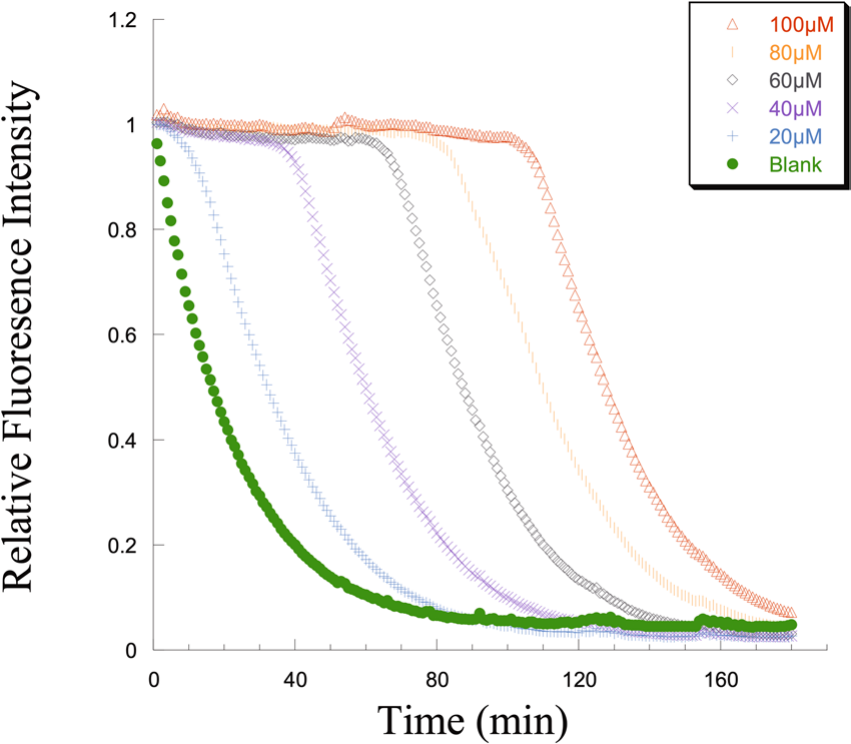
Fluorescence decay curve of fluorescein in the absence and presence of Trolox to determine Trolox’s radical scavenging capacity. The relative fluorescence intensity of fluorescein was diminished by the OH• radical reactive oxygen species. Trolox is a free radical scavenging compound that removes OH• groups from the solution and returns the fluorescence intensity of fluorescein in a dose dependent manner. The relative fluorescence intensity lasted for ~10, 50, 80, 100 and 120 min in presence of 20, 40, 60, 80, and 100 μΜ Trolox, respectively.

Trolox serves as a potent scavenger of OH• radicals, efficiently blocks the effects of highly reactive free radicals even at low concentration and therefore, is tested to prevent the photochemical degradation of melatonin in PolyRad nanoparticulate form. Fluorometric Hydroxyl Radical Scavenging Capacity (HOSC) was performed to analyze the OH• scavenging capacity of Trolox in its free solution. Results showed that an increase in Trolox concentration in the HOSC medium increased the free radical scavenging capacity of Trolox. Figure 5 shows the fluorescence decay curve of fluorescein with time in the presence of different Trolox concentrations. In the absence of Trolox (Blank), the fluorescence intensity of fluorescein was quenched rapidly with more than 50% reduction in the relative fluorescence intensity within 10 min, indicating the reaction of fluorescein with OH• radicals caused by the Fenton like Fe^3+^/H_2_O_2_ system. Trolox worked as OH• radical scavenging agent that delayed the decay in fluorescence intensity of fluorescein by diminishing the quenching rate significantly in a dose-dependent manner. As the Trolox concentration was increased from 20 to 100 μM, the capacity for scavenging OH• radicals increased. The ~100% fluorescence intensity retention time were 10, 50, 80, 100 and 120 min using 20, 40, 60, 80 and 100 μM Trolox, respectively.

### Effects of photochemical degradation on PolyRad

**Figure 6:**
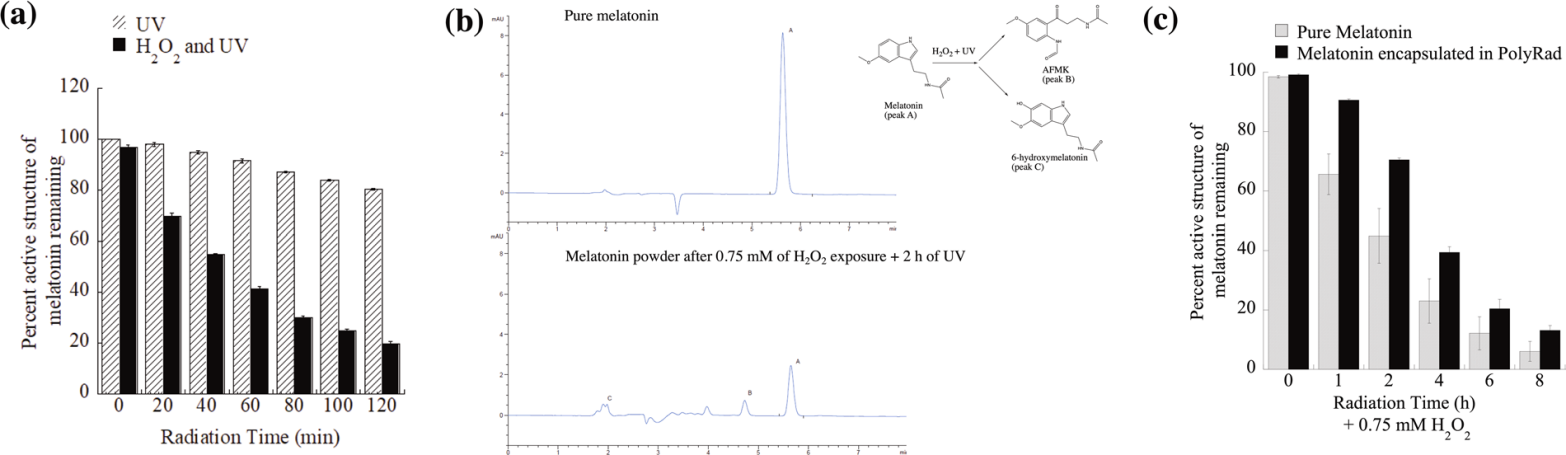
PolyRad protects melatonin from photochemical degradation. (**a**) 2.5 μg melatonin was spiked with 0.75 mM H_2_O_2_ followed by UV irradiation (254 nm) every 20 min for 2 h. Samples were freeze-dried, dissolved in 1 ml of 40% acetonitrile-60% water and analyzed using HPLC. The column graphs show the percent active structure of melatonin remaining after UV radiation only (hatched, open columns) and after H_2_O_2_ and UV treatments (black, filled columns). **(b)** The changes in melatonin peak areas after H_2_O_2_ and UV treatments. **(c)** PolyRad (black columns) was exposed to 0.75 mM H_2_O_2_ and UV treatments for longer exposure time for up to 8 h to find what percentage of melatonin is protected by PolyRad compared to bare melatonin (hatched columns). The columns represent mean of n = 3 and error bars denote standard deviation (SD).

To study the effect of photochemical oxidation on the structure of melatonin in PolyRad formulation, irradiation experiments were performed on PolyRad suspensions using H_2_O_2_ in 40% acetonitrile-60% water solvent followed by UV light exposure. Bare melatonin was used as a control. This process relies on the production of OH• through the irradiation of H_2_O_2_ with UV radiation: ${H}_{2}{O}_{2}\mathop{\to }\limits^{h\nu }2{\rm{OH}}\bullet$ that degrades melatonin^12,15^. The influence of H_2_O_2_ and UV on melatonin degradation was analyzed with time using HPLC (Fig. 6a). The melatonin peak did not change by more than ~20% by UV treatment only for 2 h (Fig. 6a; hatched column). UV irradiation in the presence of H_2_O_2_ influenced the disappearance of the melatonin peak by ~80% within 2 h (Fig. 6a; filled column). Pure melatonin control showed only one characteristic peak with a retention time of 5.67 min eluted from the HPLC column (Fig. 6b**)**. The photochemical degradation of melatonin (N-acetyl-5-methoxytryptamine) formed two major degradant peaks (B and C) at 4.7- and 1.98-min retention times for *N*^1^-acetyl-*N*^2^-formyl-5-methoxykynuramine (AFMK) and 6-hydroxymelatonin, respectively^4,59–61^. The measured changes of AFMK and 6-hydroxymelatonin concentrations increased as a function of different UV exposure time in the presence of H_2_O_2_ (SI Figs. 2–4). Also, a peak at ~ 3.95 min, likely due to the melatonin degradation product, N-acetyl-5-methoxykynuramine (AMK) is seen. The overall reaction followed a first-order reaction: $-{r}_{{Melatonin}}=k{C}_{{melatonin}}$, where $-{r}_{{Melatonin}}$, $k$ and ${C}_{{melatonin}}$ represent the rate of disappearance of melatonin, reaction rate constant and concentration of melatonin, respectively (SI Fig. 5). The reaction rate constant ($k$) is determined as $(8\times {10}^{-5}){{\min }}^{-1}$ (SI Fig. 5a). In contrast to bare melatonin (Fig. 6c, hatched column), a remarkable reduction of melatonin degradation was observed using PolyRad nanoparticles (Fig. 6c, filled column**)**. PolyRad protected ~75, 65, 55 and 15% of the active structure of melatonin after UV irradiation only, and 2 h of UV followed by 0.75, 1, and 5 mM of H_2_O_2_ exposure, respectively indicating that nanoparticles scavenged H_2_O_2_ in a concentration-dependent manner. Encapsulation of melatonin using PolyRad nanoparticles reduced the formation of AFMK by ~50% (SI Fig. 6).

### ^1^H-NMR spectrometry studies on molecular structure of melatonin after gamma radiation

**Figure 7:**
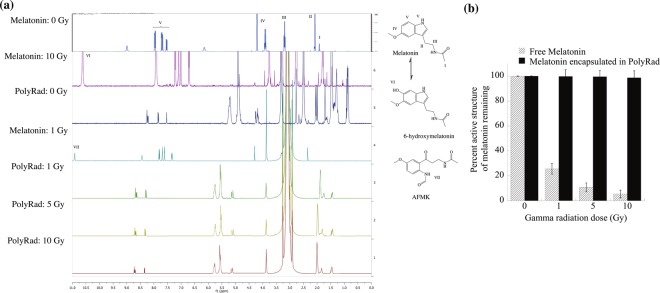
The effects of gamma irradiation on melatonin using ^1^H-NMR spectroscopy. (**a**) Melatonin degradation was observed at 1, 5 and 10 Gy doses using a Co-60 source. The structure of melatonin alone without radiation is shown as a control. **(b)** The areas under the curve of ^1^H NMR peaks were integrated to determine the percentage melatonin degradation. Experiments were performed at least in triplicate to estimate the mean and standard deviation of each peak area.

Based on the ^1^H NMR spectrum of melatonin as presented in Fig. 7a and Table 2, major melatonin peaks are observed at 1.9 (NHCOCH_3_), 2.9 (CH_2_), 3.5 (CH_2_NH), 3.9 (OCH_3_) and 6.9–7.4 ppm (benzene aromatic rings and NHCHO). Free melatonin is degraded into other compounds that appear at 3.2, 3.6 and 3.78 ppm and predominantly at 10.6 (OH•) and 10.9 (NHCO) ppm. Melatonin in PolyRad nanoparticles did not show any significant differences between the non-radiated control and gamma irradiated PolyRad samples. The percentage of active melatonin remaining was calculated using the integral ratio of peaks of groups I through V (Table 2) based on the non-radiated melatonin control (Fig. 7b). The structural integrity of melatonin alone was completely degraded to 25.7, 10.8 and 5.5% at 1, 5 and 10 Gy, respectively compared to non-radiated free melatonin. The nanoparticle plays a critical role in the protection of melatonin by >98% most likely by scavenging of free radicals generated from gamma radiation^62^.

**Table 2:** Identification of ^1^H NMR peaks of the molecular structure of melatonin after Co-60 gamma radiation.

Peak center (ppm)	1.9	2.9	3.5	3.9	6.9–7.4	10.6	10.9
**Symbol**	I	II	III	IV	V	VI	VII
**Chemical identity**	-NHCOCH_3_	-CH_2_	CH_2_NH	OCH_3_	C_6_H_6_; NHCHO	OH•	-NHCO

### ***I****n vitro***drug release kinetics**

**Figure 8:**
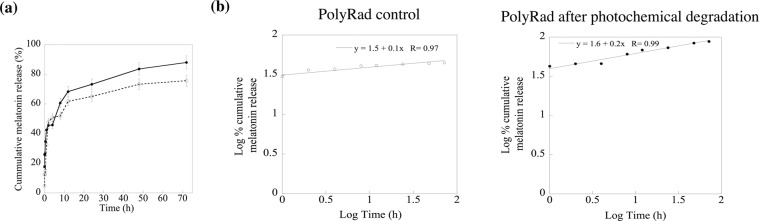
*In vitro* release profiles of melatonin from PolyRad. (**a**) *In vitro* release curves of melatonin from PolyRad in PBS of pH 7.4 at 37 °C before (open circle, dotted line) and after the photochemical degradation (solid circle, solid line) of PolyRads. **(b)** Melatonin release data were fitted to power law models before and after photochemical degradation of PolyRads. The column bar represents the mean value with standard deviations for n = 3 independent experiments.

The melatonin release kinetics from PolyRad is shown in Fig. 8a before and after radiation. A total of 70% melatonin was released from non-radiated nanoparticles after 72 h at pH 7.4. Notably, more than 50% of the drug was released after only 2 h, suggesting a spontaneous rapid degradation of solid nanoparticles under physiological conditions. Exposure to radiation elicited higher drug release, reaching >90% drug release in 72 h. The drug release profile follows a biphasic pattern, indicating a controlled drug release mechanism. The data from *in vitro* experiments were fitted to a power-law model to investigate the kinetics of melatonin release (Fig. 8b and Table 3). The cumulative release is linear with respect to time, $t$ (${R}^{2} > 0.97$) where diffusion exponent $n$ varies between 0.1–0.3, indicating a Fickian diffusion profile. The rate constant ($k$) varies between 0.3–0.4 suggesting a slow melatonin release from PolyRad.

**Table 3:** Kinetic modeling of melatonin release from PolyRad nanoparticles.

PolyRad	Model Equation	*R*^2^	Rate constant, *k*	Diffusion exponent, *n*
Non-radiated	*y* = 1.5 + 0.1*x*	0.96	0.32	$0.1$
Photochemical irradiation	*y* = 1.6 + 0.2*x*	0.98	0.40	$0.2$

### **C**ytotoxicity assay

**Figure 9:**
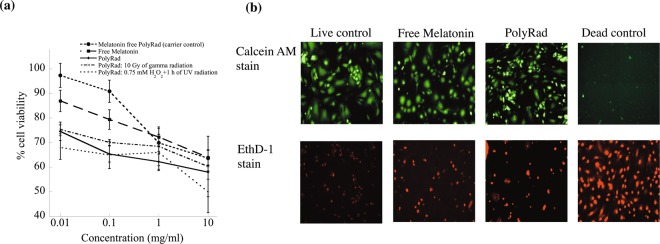
Effect of melatonin on HUVEC cell viability. (**a**) MTT assay shows that PolyRad is non-toxic at concentrations of $\le$ 0.01 mg/ml. Data represents mean and standard deviation of three independent experiments. **(b)** Calcein AM (green fluorescence) and EthD-1 (red fluorescence) live/dead assay stain live and dead HUVEC, respectively after 0.01 mg/ml of PolyRad incubation.

The effects of melatonin solution, PolyRad without melatonin and PolyRad (with melatonin) on the viability of HUVEC were analyzed using MTT assay and live/dead assay imaging (Fig. 9). As the concentration of melatonin in PolyRad (♦) increases, cell viability decreases from 75% to ~60% at melatonin concentrations ranging from 0.01 to 10 mg/ml. Free melatonin (■) showed ~90% cell viability at 0.01 mg/ml which dropped to ~65% at 10 mg/ml. Independent radiation treatments using gamma (**×**) and UV irradiation (**+**) after H_2_O_2_ exposure showed similar effects on cell viability as non-radiated PolyRad, indicating radiation did not affect the release of melatonin from the nanoparticles. Melatonin free PolyRad (•) had no significant toxicity at 0.01 and 0.1 mg/ml, the cell viability dropped to ~65% at 10 mg/ml. Results show an increased melatonin cytotoxicity in the order of free melatonin, non-irradiated PolyRad and irradiated PolyRad. We further studied if melatonin and PolyRad interfered with the cell viability of HUVEC. As shown in Fig. 9b, live cells after treated with 0.01 mg/ml each of melatonin and PolyRad fluoresce green as stained with calcein AM. Cells show a slight cytotoxicity as assessed by dead cell staining (red fluorescence) using EthD-1.

## Discussion

Melatonin is an endogenous neurohormone that regulates many biological functions such as sleep, circadian rhythm, immune responses and cardiac functions, among others^63–66^. Changes of environment in spaceflight compared to that on Earth disrupt sleep and circadian clock in astronauts’ physiology decreasing the performance^1,67,68^. Sleep medications such as melatonin are used to improve astronauts’ circadian rhythms and sleep^4^. Space medication is a challenge because of the instability of active pharmaceutical ingredients (API) in the presence of harsh spaceflight environmental conditions. Altered physical and chemical stability in pharmaceuticals can result in reduced potency which can result in reduced efficacy. It has been tested that the API of melatonin degrades in the adverse environments of space station^4,60^. The traditional approach to overcome the limitation is to replace medicines from the International Space Station (ISS) before their expiration. However, this is not a practical solution for longer duration missions to planets (*e.g*., Mars). Hence, it is desired that medicines maintain their shelf-life throughout the duration of the space mission. Contrary to standard dispensing practices on Earth, pharmaceuticals are packed and dispensed in special flight-certified containers and stored inside compactly packed kits. The packaging could affect the active pharmaceutical ingredients and therapeutic efficacy of medications in space^60,69^. The shielding material should meet the requirement of application in radiotherapy, should be light-weight, flexible, environmental friendly and easily recyclable. According to these requirements, there is a need to provide effective shielding against space medications with materials of appropriate designs that can maintain the stability of space medicines.

Our results show that antioxidant conjugated polymeric nanoparticles (PLGA-PLL-Trolox) can act as an effective and protective shield for pharmaceuticals that are being exposed to high energy radiations in space. Trolox antioxidant effectively acts as a radiation-induced highly reactive OH• scavenger that protects the pharmaceuticals prolongating the shelf-life and thus the potency. Trolox can scavenge free radicals through a variety of reaction mechanisms, as it is the case for many other scavengers^62^. Four reaction mechanisms have been reported to significantly contribute to the OH• scavenging capacity of Trolox in aqueous solution: hydrogen transfer (HT), radical adduct formation (RAF), single electron transfer (SET) and sequential proton loss electron transfer (SPLET)^62^. The free radical scavenging capacity of Trolox has been shown to reduce oxidative stress in aqueous systems as well as epithelial cells, hepatic injury and red blood cells *in vitro* and *in vivo*^70–74^. Fluorescein, organic compound used in this assay, has characteristic of high photostability, stability at physiological pH and is highly sensitive to OH• attacks. Accordingly, Trolox protected fluorescein from OH• attacks by scavenging the radicals, prolonging the relative fluorescein intensity. Figure 5 shows that conjugation of Trolox to the surface of drug-loaded PolyRad leads to successful shielding of encapsulated pharmaceuticals from the damaging effects of photochemical degradation. When PolyRads were photochemically degraded using UV light and H_2_O_2_, part of their covalent bonds dissociated which in turn protected the active structure of melatonin (Fig. 6c). AFMK and 6-hydroxymelatonin appear to be the two main degradants of pure melatonin which is blocked in PolyRad formulations. The antiradical activity of PolyRad was further detected using gamma radiation and analyzed *via* ^1^H NMR showing>98% protection of active melatonin structure in nanoparticles (Fig. 7a,b).

*In vitro* drug release follows a biphasic kinetics pattern. The *in vitro* release for non-irradiated PolyRad showed a burst release of ~20% of the encapsulated melatonin (Fig. 8a). The initial burst release decreased after photochemical degradation. The polymer coating began to disintegrate, releasing ~65% and 75% of total melatonin from non-radiated and irradiated PolyRad, respectively after 24 h and ~70% and 85% from non-radiated and irradiated PolyRad, respectively after 72 h. The exponent, n, characteristic of the overall mechanism of drug release increased after photochemical degradation (Fig. 8b). The values of the exponent of time, n, indicating that the release of melatonin (high diffusivity) is at least partially controlled by the scission of the polymer. The *in vitro* data provide guidance for understanding the fundamentals of melatonin drug release. *In vitro* cell culture studies indicate that melatonin and PolyRad induce cytotoxicity to HUVEC in a dose-dependent manner (Fig. 9a)^75,76^. Generation of reactive oxygen species has been reported to promote apoptosis in HUVEC while maintaining normal redox homeostasis^77,78^. It is possible that PolyRad increases ROS production suggesting higher cell death at concentrations above 0.01 mg/ml (10 μg/ml). The ability of PolyRad to release melatonin was not affected after irradiation. PolyRad is as potent as free melatonin in eliciting therapeutic effects in HUVEC growth (Fig. 9b).

## Conclusion

Here, we have described a new technology for protection of pharmaceuticals against radiation induced damages (*i.e*., free radical exposure), which is likely to occur during space missions. We synthesized PolyRad nanoparticles, 325 ± 21 nm in hydrodynamic diameter, using the emulsion solvent evaporation method by encapsulating melatonin within the nanoparticles to protect APIs from radiation damage. Conjugation of Trolox to the surface of PLGA nanoparticles significantly inhibited the photochemical degradation of active melatonin. Nonetheless, the current results are promising and continue to shed light on the inherent benefits of Trolox as an antioxidant that acts as a free radical scavenger to shield spaceflight pharmaceuticals from the deleterious effects of incident radiation and helps maintain their shelf-life and potency. PolyRad is demonstrated to be particularly useful for shielding space pharmaceuticals from the toxic effects of space radiation as induced by free radicals with the conjugation of antioxidants on the surface of the nanoparticles. Results from this research can be further extended to cancer treatment by reducing oxidative stress and preventing the damage of normal cells caused by high-energy radiation therapy, which is used to shrink tumors and kill cancer cells.

## Supplementary information

Supplementary information.
