# Supplementary material for: PolyRad – Protection Against Free Radical Damage
Source: Sci Rep. 2020 May 20;10:8335. doi: 10.1038/s41598-020-65247-y (PMC7239908; doi:10.1038/s41598-020-65247-y)
Supplement: Supplementary file 1 — Supplementary information. [file 41598_2020_65247_MOESM1_ESM.pdf]

Supplementary Information

PolyRad – Protection Against Free Radical Damage

Hannah Kim,[a*] Yin Tse,[b†] Andrew Webb,[b‡] Ethan Mudd,[b§] Muhammad Raisul Abedin,[b] Melanie Mormile,[a] Shubhadeep Dutta,[c] Kaushal Rege,[d] and Sutapa Barua[**, b]

[a] Department of Biological Sciences

[b] Department of Chemical and Biochemical Engineering

Missouri University of Science and Technology, Rolla, MO 65409

[c] School of Molecular Sciences

Arizona State University, Tempe, AZ 85287

[d] Chemical Engineering, School for Engineering of Matter, Transport and Energy

Arizona State University, Tempe, AZ 85287

[*] The authors contributed equally
[†] The authors contributed equally
[‡] The authors contributed equally
[§] The authors contributed equally
[**] To whom correspondence should be addressed. Email: baruas@mst.edu. Department of Chemical & Biochemical Engineering, 110 Bertelsmeyer Hall, 1101 N. State Street, Rolla, MO 65409-1230

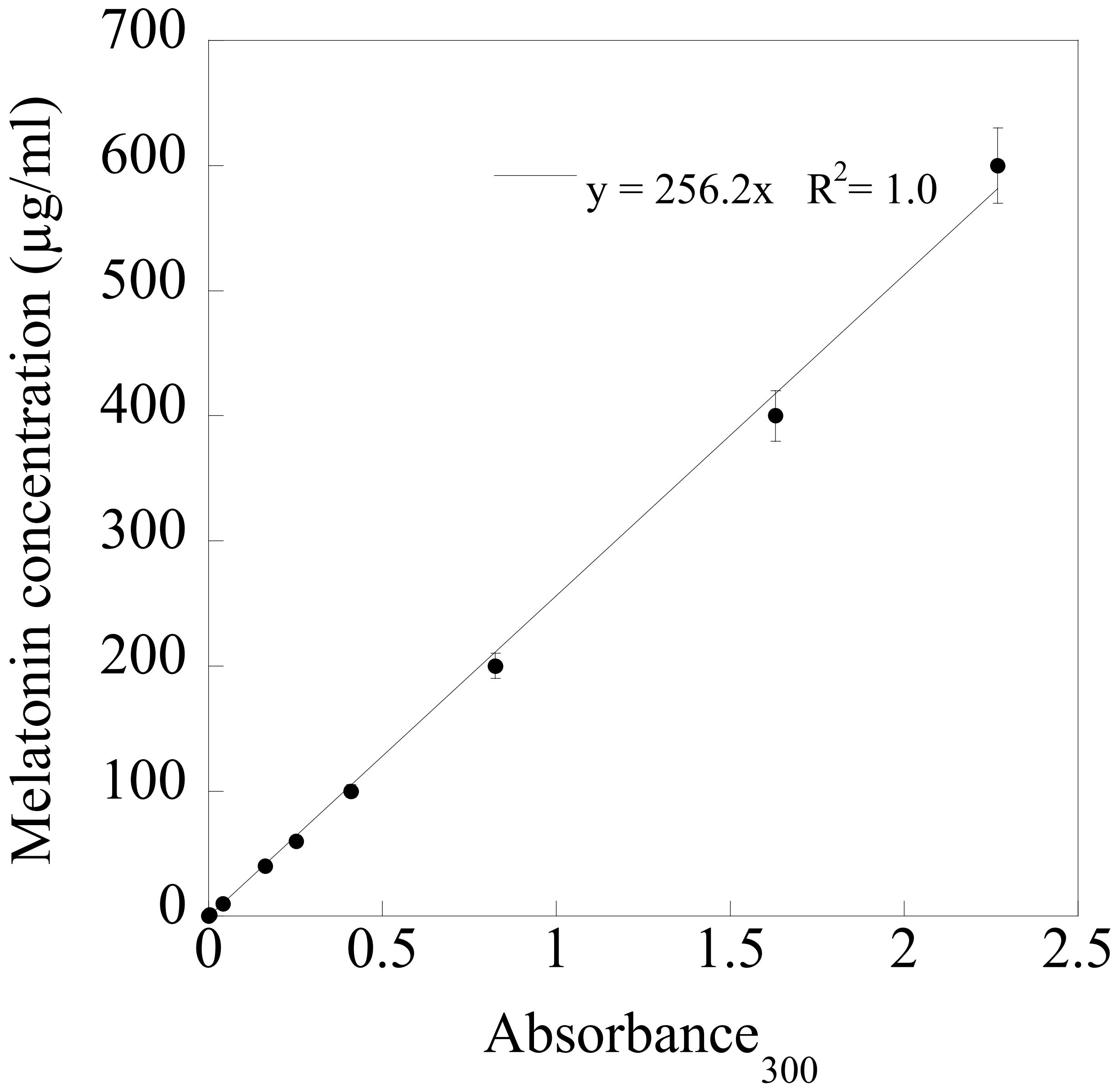

SI Figure 1: Calibration curve of melatonin that is used to calculate the concentration of melatonin of unknown concentrations.

Melatonin powder after 0.75 mM of H_2O_2 exposure + 20 min of UV

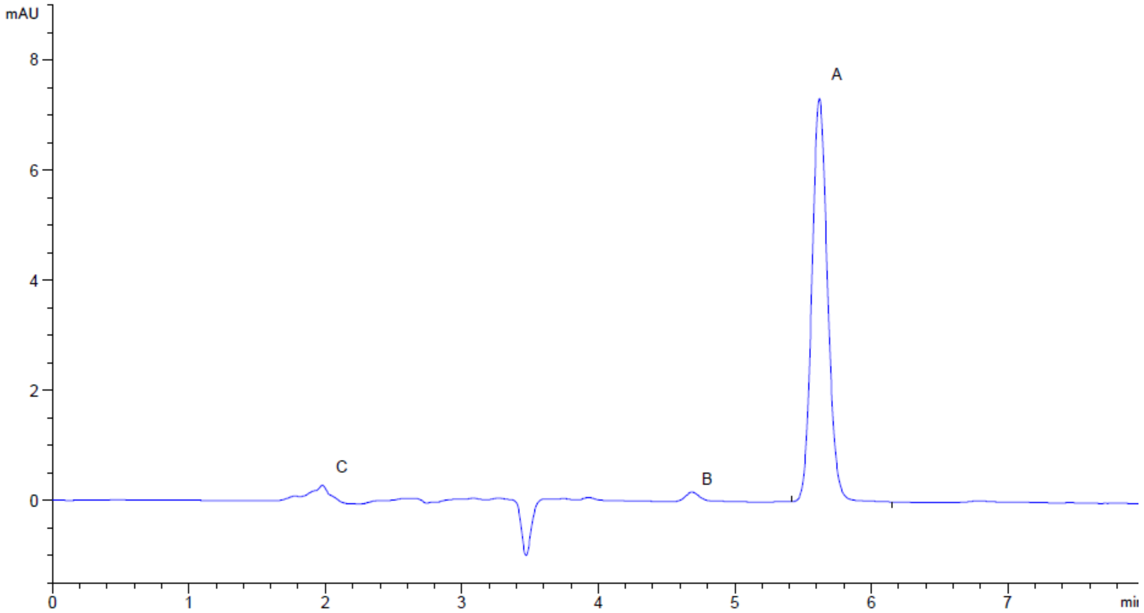

Melatonin powder after 0.75 mM of H_2O_2 exposure + 40 min of UV

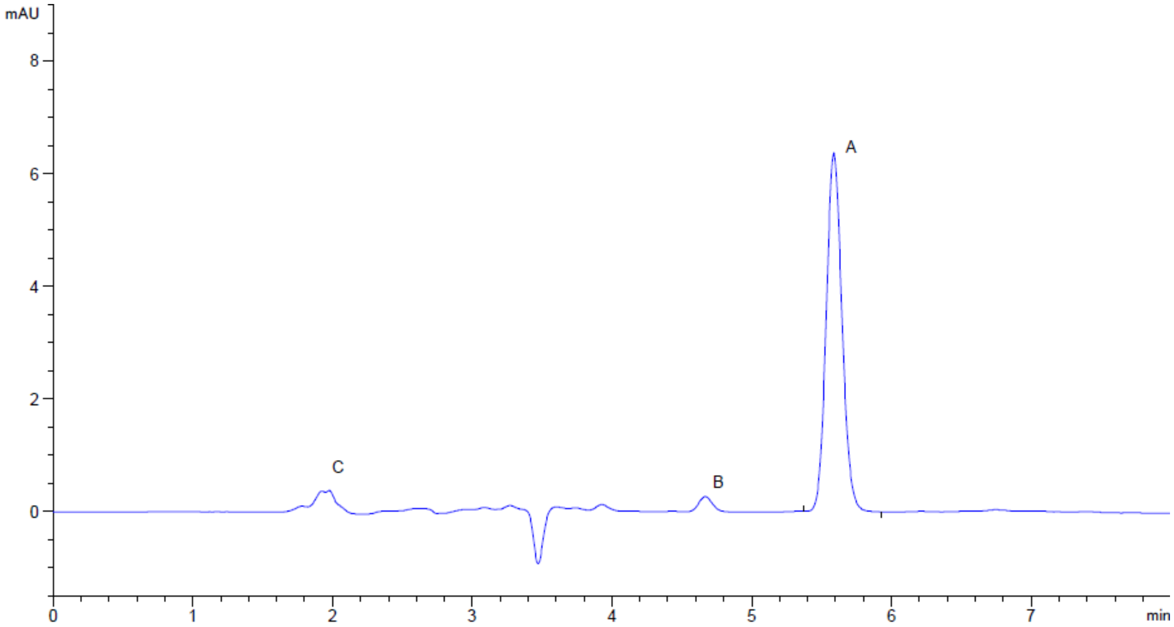

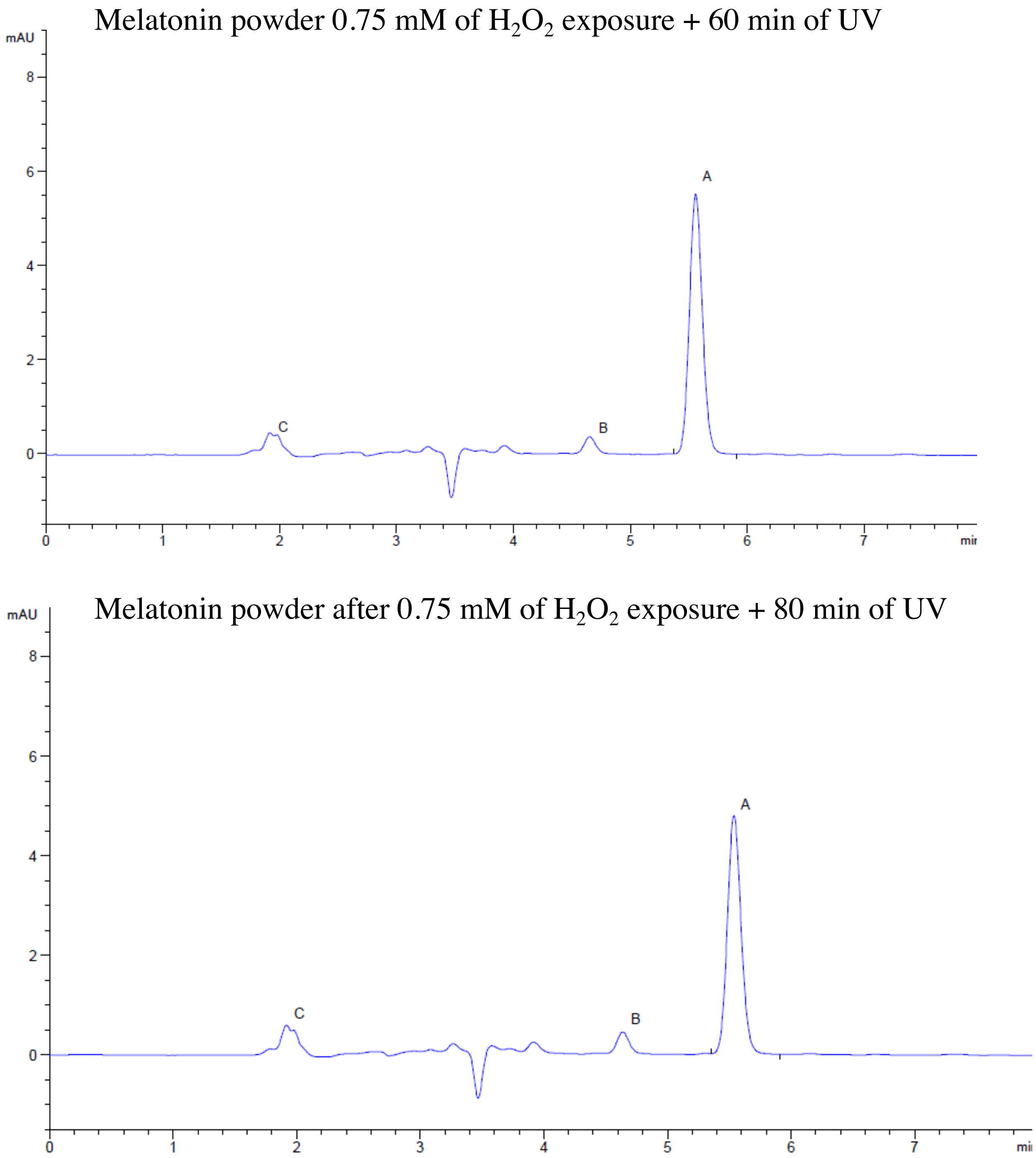

SI Figure 2: HPLC curves of pure melatonin powder after H_2O_2 exposure and UV radiation at 20 min intervals. A, B and C represent characteristic HPLC peaks of melatonin, AFMK and 6-hydroxymelatonin, respectively at ~5.5, 4.7 and 1.98 min.

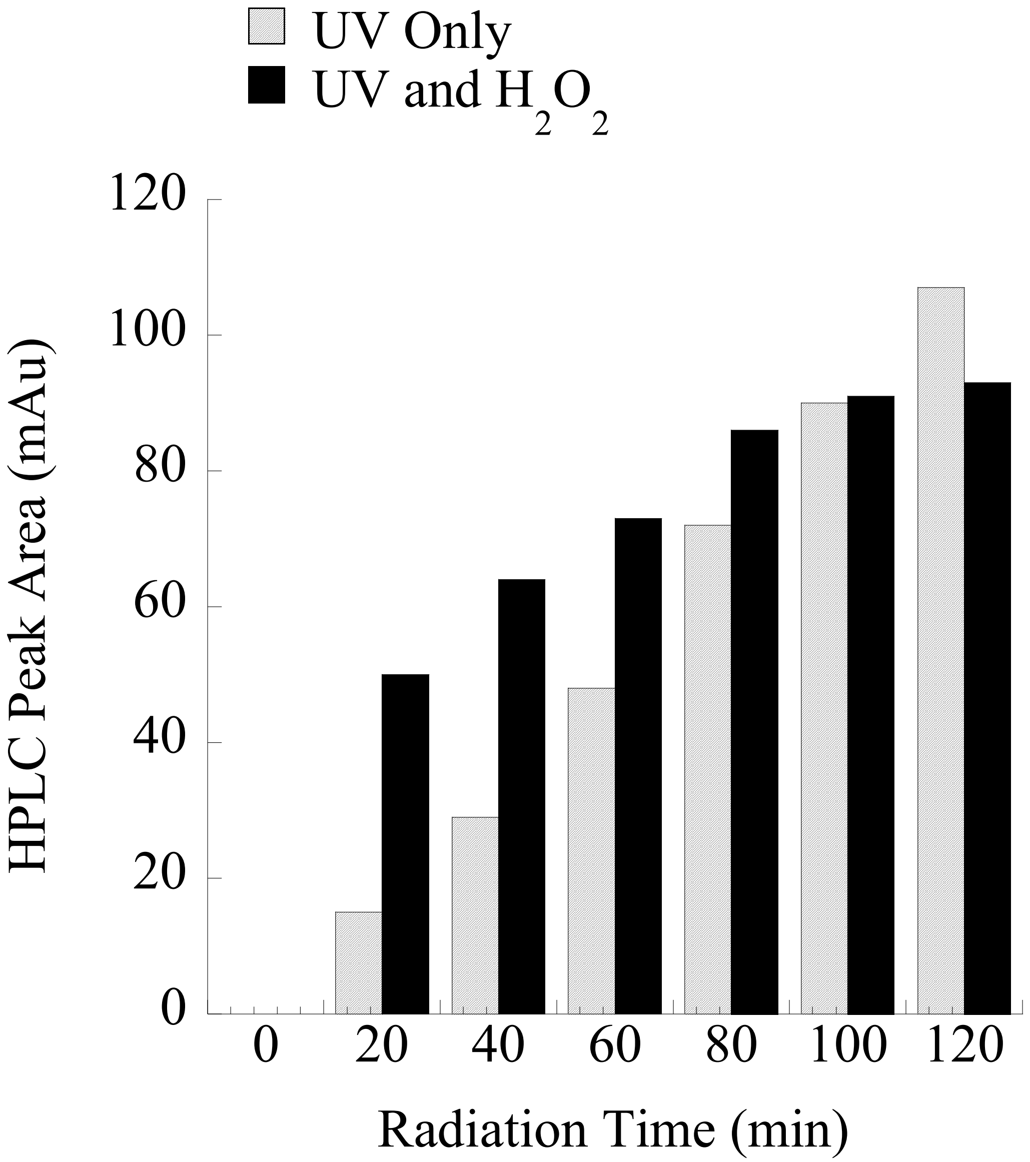

SI Figure 3: Formation of AFMK degradant (peak B SI Figure 2) from melatonin as a function of UV irradiation exposure time. Gray columns indicate UV irradiation alone control. Black columns represent the simultaneous H_2O_2 exposure and UV irradiation. Degradation of melatonin into AFMK was enhanced by simultaneous H_2O_2 exposure and UV irradiation.

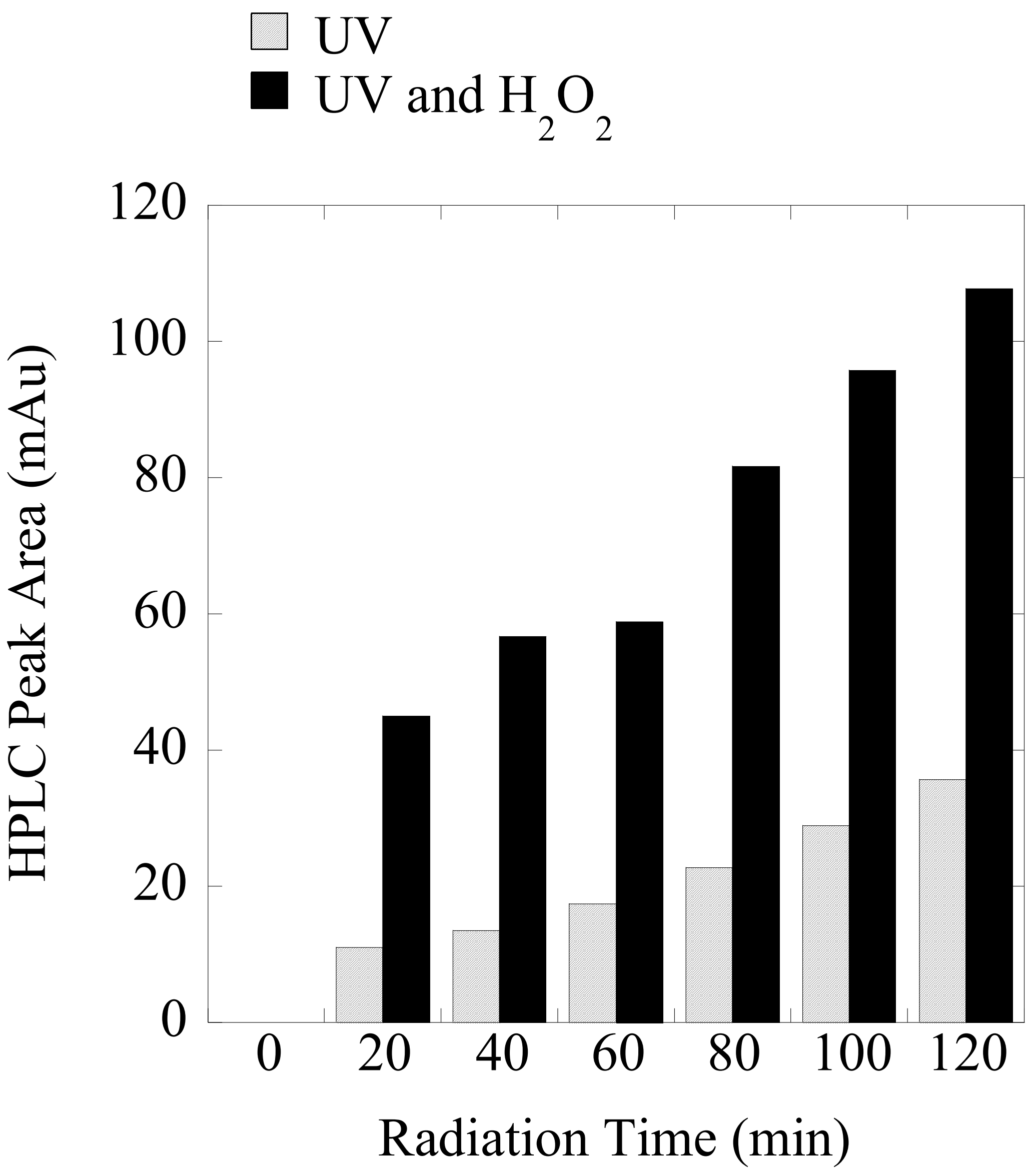

SI Figure 4: Generation of 6-hydroxymelatonin (peak C in SI Figure 2) by the degradation of melatonin using UV only (gray columns) and UV and H_2O_2 treatments (black columns).

(a)

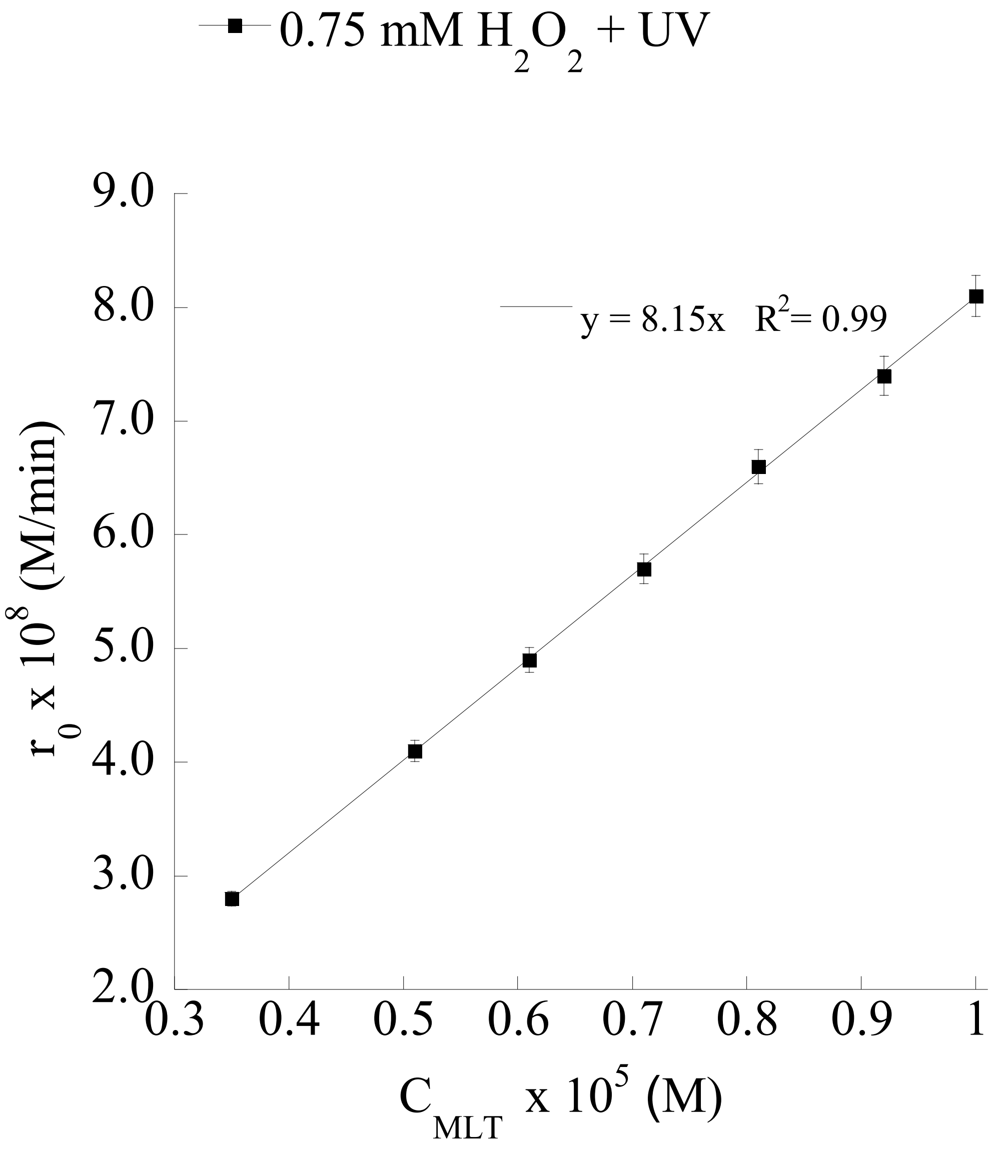

0.75 mM H2O2 + UV
y = 8.15x R2= 0.99
r0 x 10^8 (M/min)
9.0
8.0
7.0
6.0
5.0
4.0
3.0
2.0
0.3
0.4
0.5
0.6
0.7
0.8
0.9
1
CMLT x 10^5 (M)

(b)

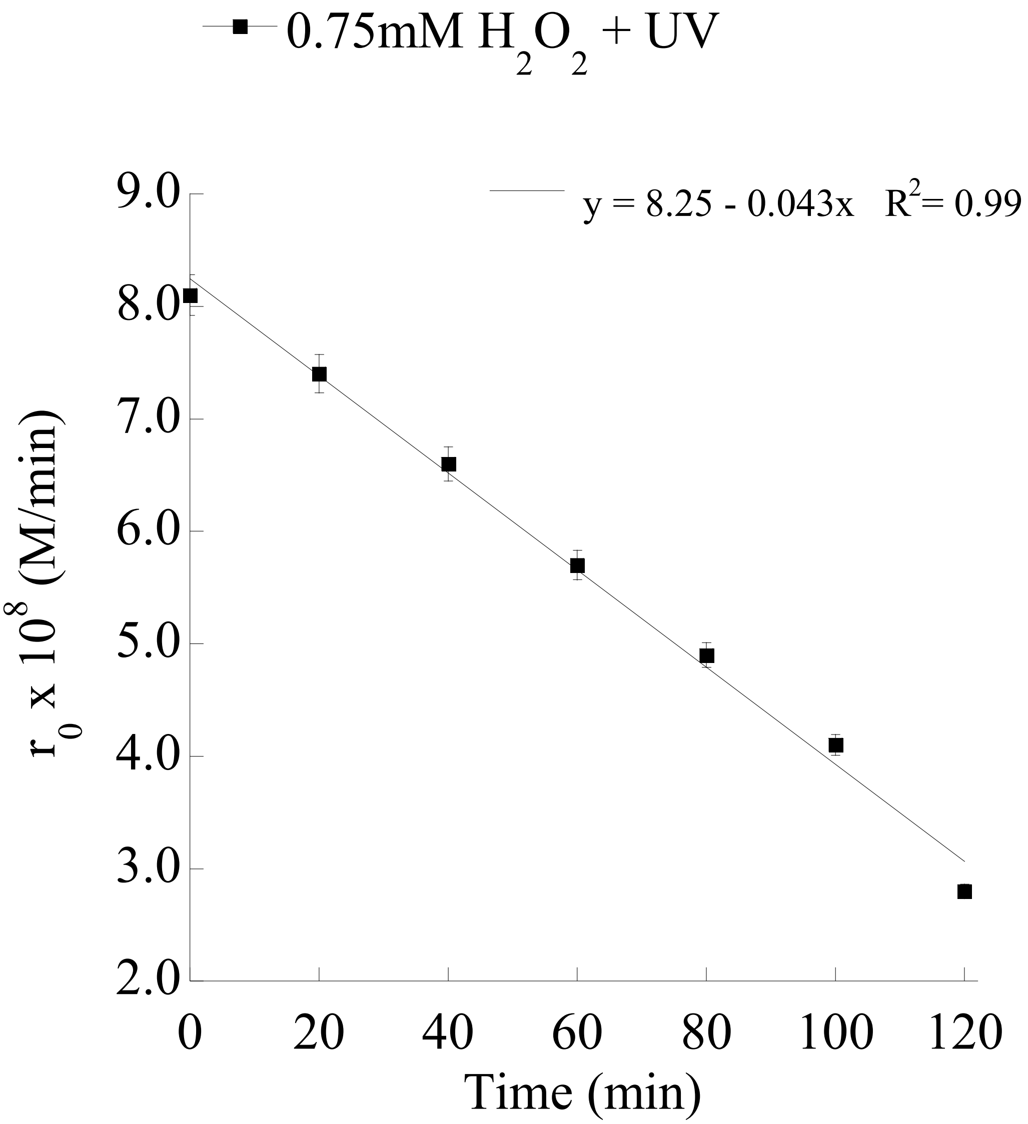

SI Figure 5: Melatonin degradation follows the first order rate law kinetics. **(a)** The experimental data of melatonin degradation rate *versus* melatonin concentration follows $-r_{melatonin} = k\ C_{mleatonin}$ reaction rate kinetics. From the linear line, the reaction rate constant, k is found $\approx 8 \times 10^{-5} M$. **(b)** The melatonin degradation rate expression as a function of irradiation time which also followed a linear correlation

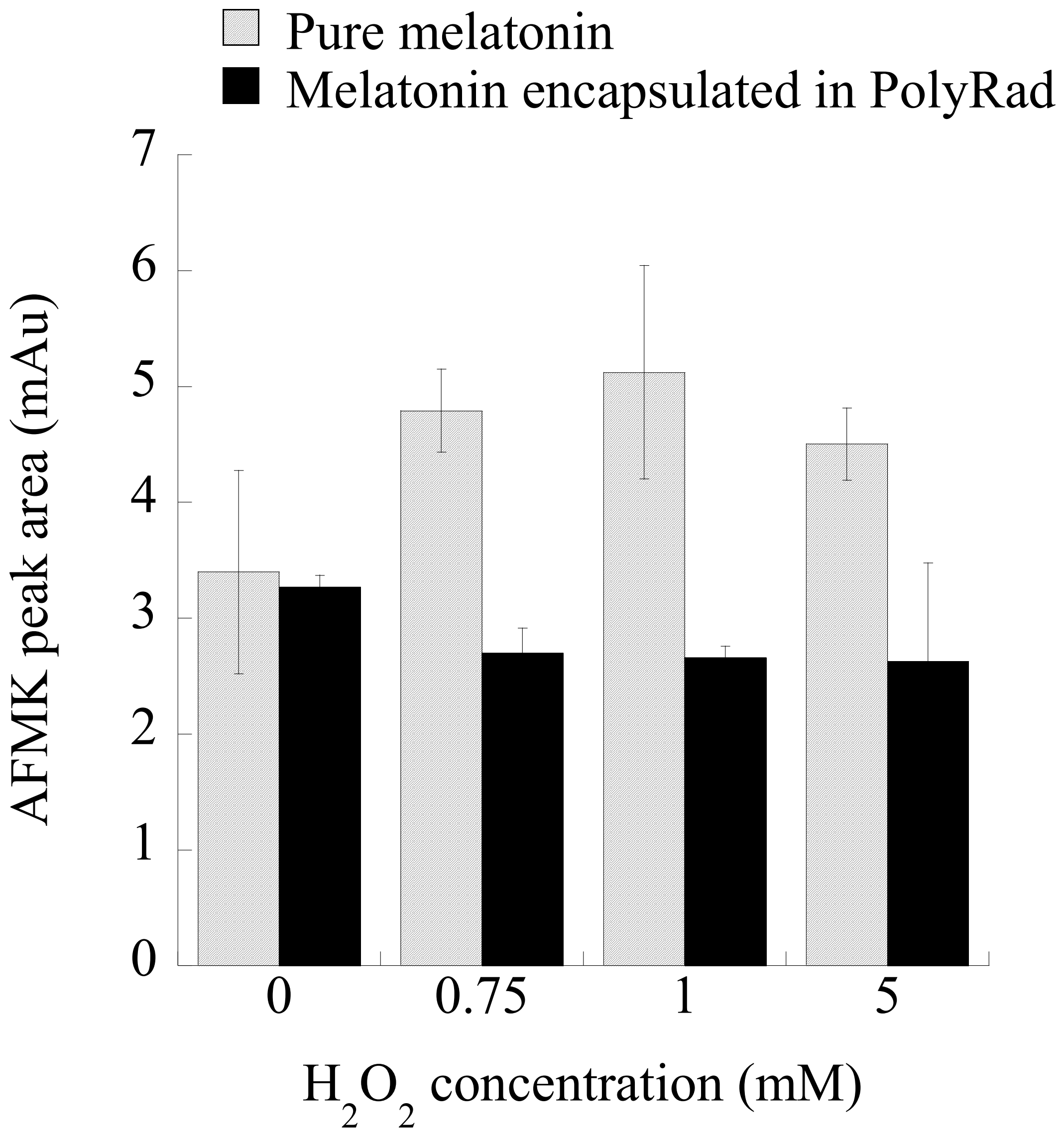

SI Figure 6: Formation of AFMK was reduced by ~50% when melatonin was encapsulated by PolyRad. 2.5 μg/ml melatonin was dissolved in 0.5 ml of 40% acetonitrile-60% water. Samples were spiked with 0, 0.75, 1 and 5 mM H_2O_2 followed by UV irradiation (254 nm) for 2 h and analyzed using HPLC. The column graphs show the percent active structure of melatonin remaining after UV radiation only (gray columns) and after H_2O_2 and UV treatments (black, filled columns). **(b)** The changes in melatonin peak areas after UV and H_2O_2 treatments.
